# Supplementary material for: Pathfinder-Driven Chemical Space Exploration and Multiparameter Optimization in Tandem with Glide/IFD and QSAR-Based Active Learning Approach to Prioritize Design Ideas for FEP+ Calculations of SARS-CoV-2 PLpro Inhibitors
Source: Molecules. 2022 Dec 5;27(23):8569. doi: 10.3390/molecules27238569 (PMC9741453; doi:10.3390/molecules27238569)
Supplement: Supplementary file 1 [file molecules-27-08569-s001.zip › molecules-2031413-supplementary.pdf]

Pathfinder-driven chemical space exploration and multiparameter optimization in tandem with Glide/IFD and QSAR-based active learning approach to prioritize design ideas for FEP+ calculations of SARS-COV-2 PL^{pro} inhibitors

N.J. Gumede*

Department of Chemistry, Mangosuthu University of Technology, PO Box 12363, Jacobs, 4026, South Africa.

Corresponding Author

*E-mail: ngumede@mut.ac.za

Supplementary Materials

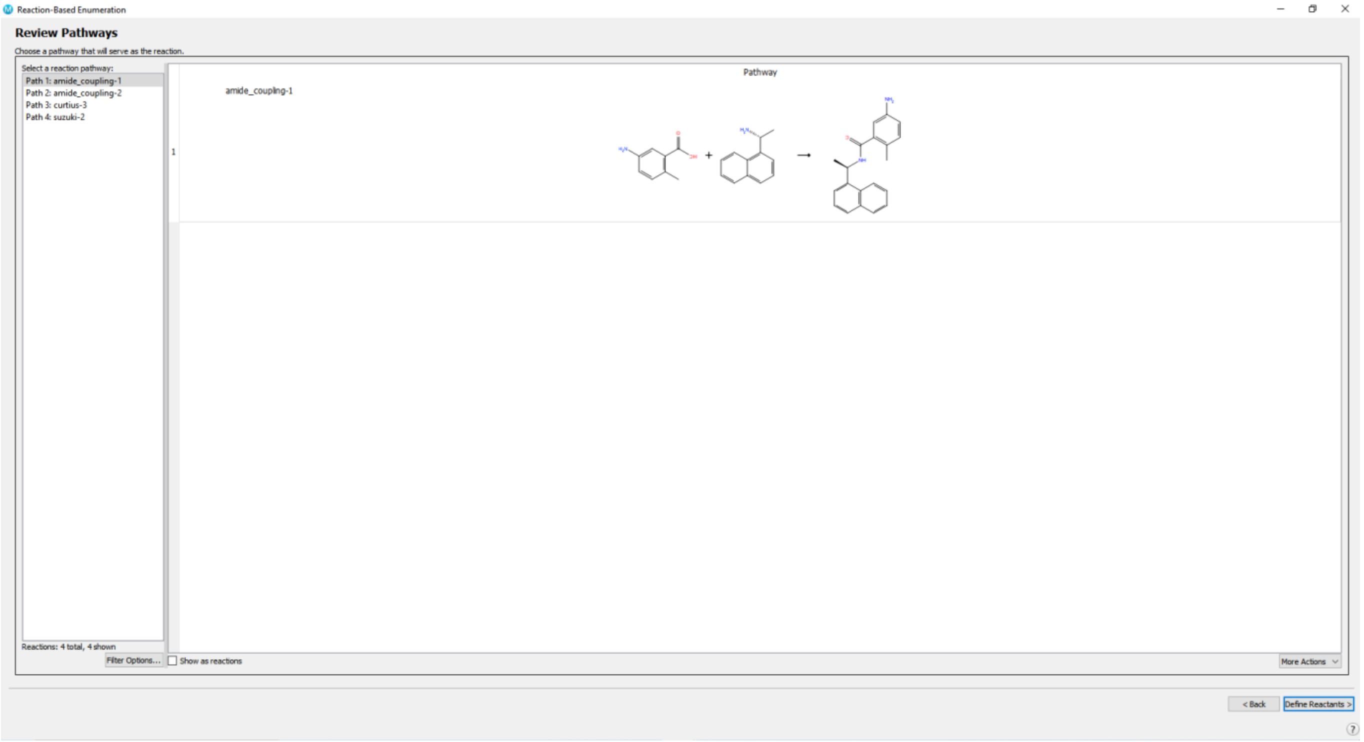

Figure S1. Reaction based enumeration generated pathway for GRL-0617.

(a)

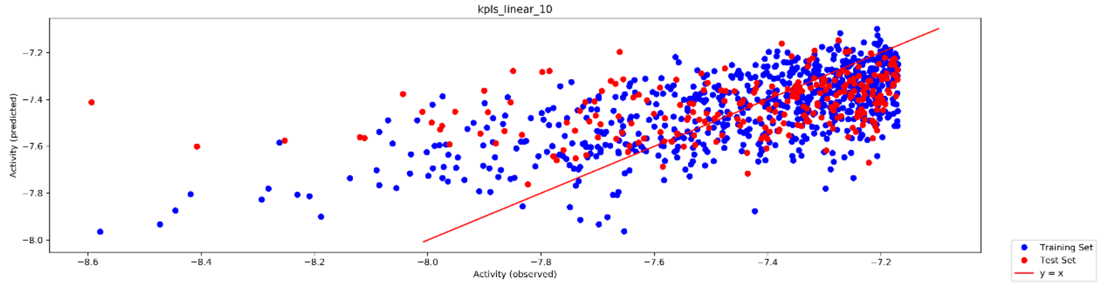
kpls_linear_10
Activity (predicted)
Activity (observed)
Training Set
Test Set
y = x

(b)

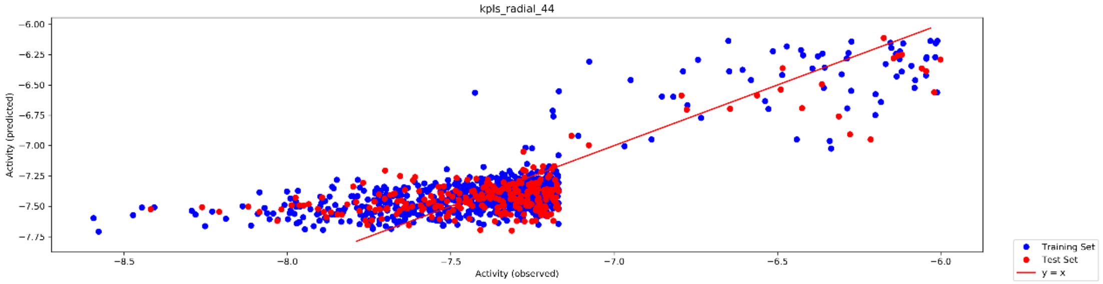
kpls_radial_44
Activity (predicted)
Activity (observed)
Training Set
Test Set
y = x

(c)

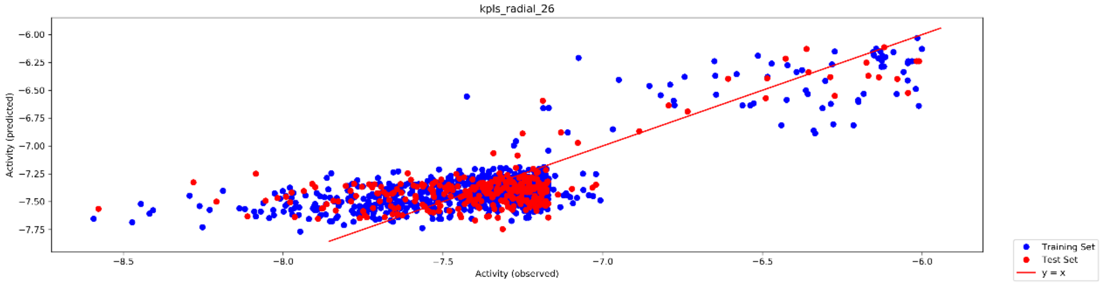
kpls_radial_26
Activity (predicted)
Activity (observed)
Training Set
Test Set
y = x

(d)

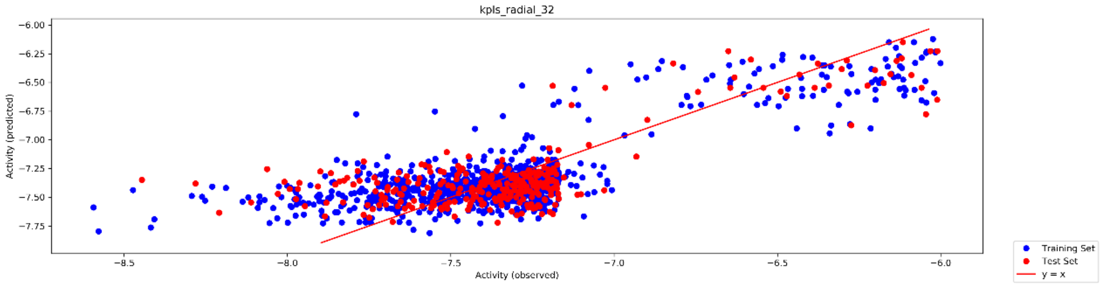
kpls_radial_32
Activity (predicted)
Activity (observed)
Training Set
Test Set
y = x

(e)

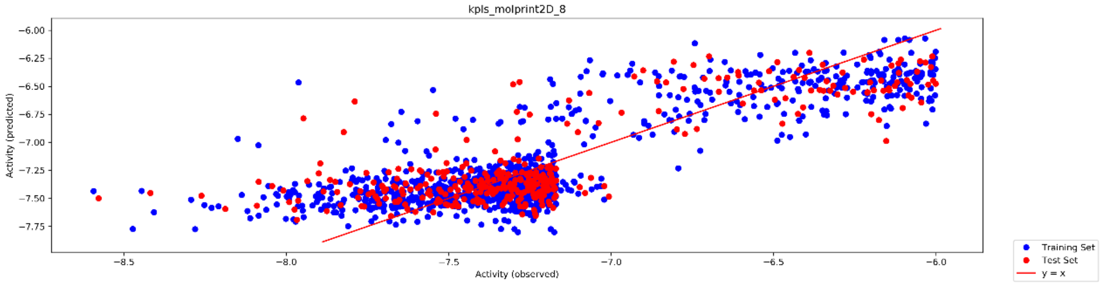

(f)

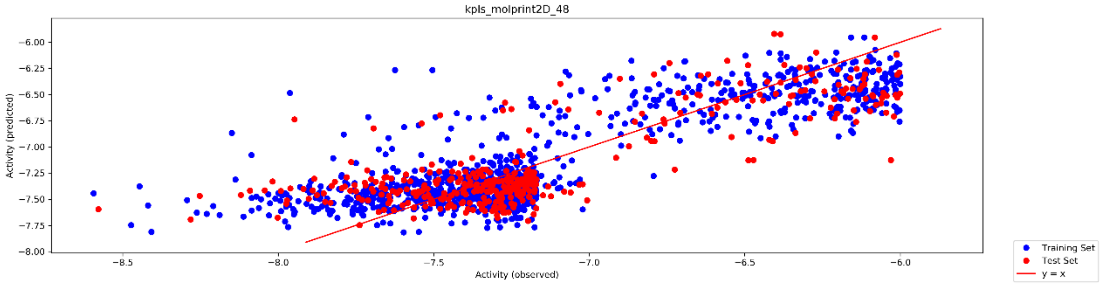

(g)

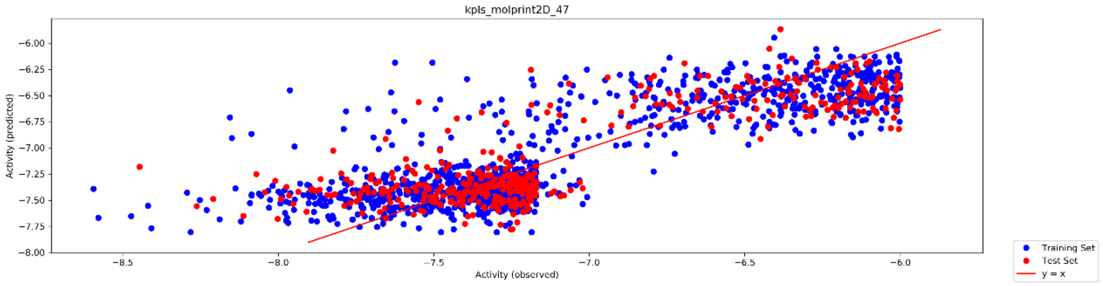

(h)

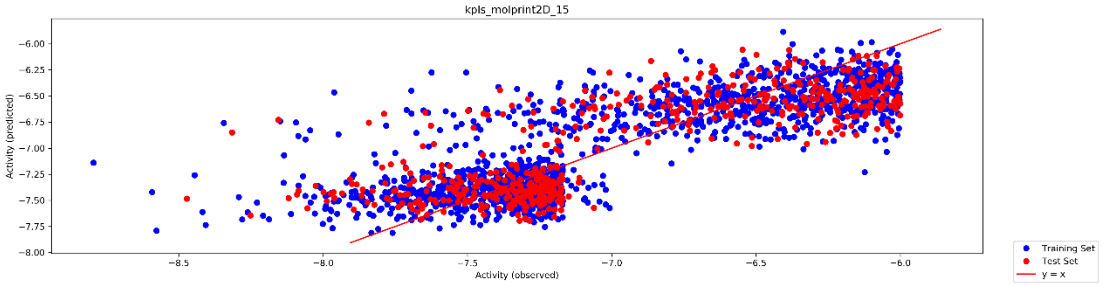

Figure S2. Plot of Active learning Glide SP and Auto QSAR models to predict the binding affinities of enumerated compounds **(a)** Auto QSAR_model_1 with an R^2 of 0.4310 and a Q^2 of 0.1571 **(b)** Auto QSAR_model_2 with an R^2 of 0.6262 and a Q^2 of 0.5929 **(c)** Auto QSAR_model_3 with an R^2 of 0.6090 and a Q^2 of 0.5630 **(d)** Auto QSAR_model_4 with an R^2 of 0.6227 and a Q^2 of 0.5793 **(e)** Auto QSAR_model_5 with an R^2 of 0.6980 and a Q^2 of 0.6450 **(f)** Auto QSAR_model_6 with an R^2 of 0.7084 and a Q^2 of 0.6676 **(g)** Auto QSAR_model_7 with an R^2 of 0.7086 and a Q^2 of 0.6910 **(h)** Auto QSAR_model_9 with an R^2 of 0.6718 and a Q^2 of 0.6476.

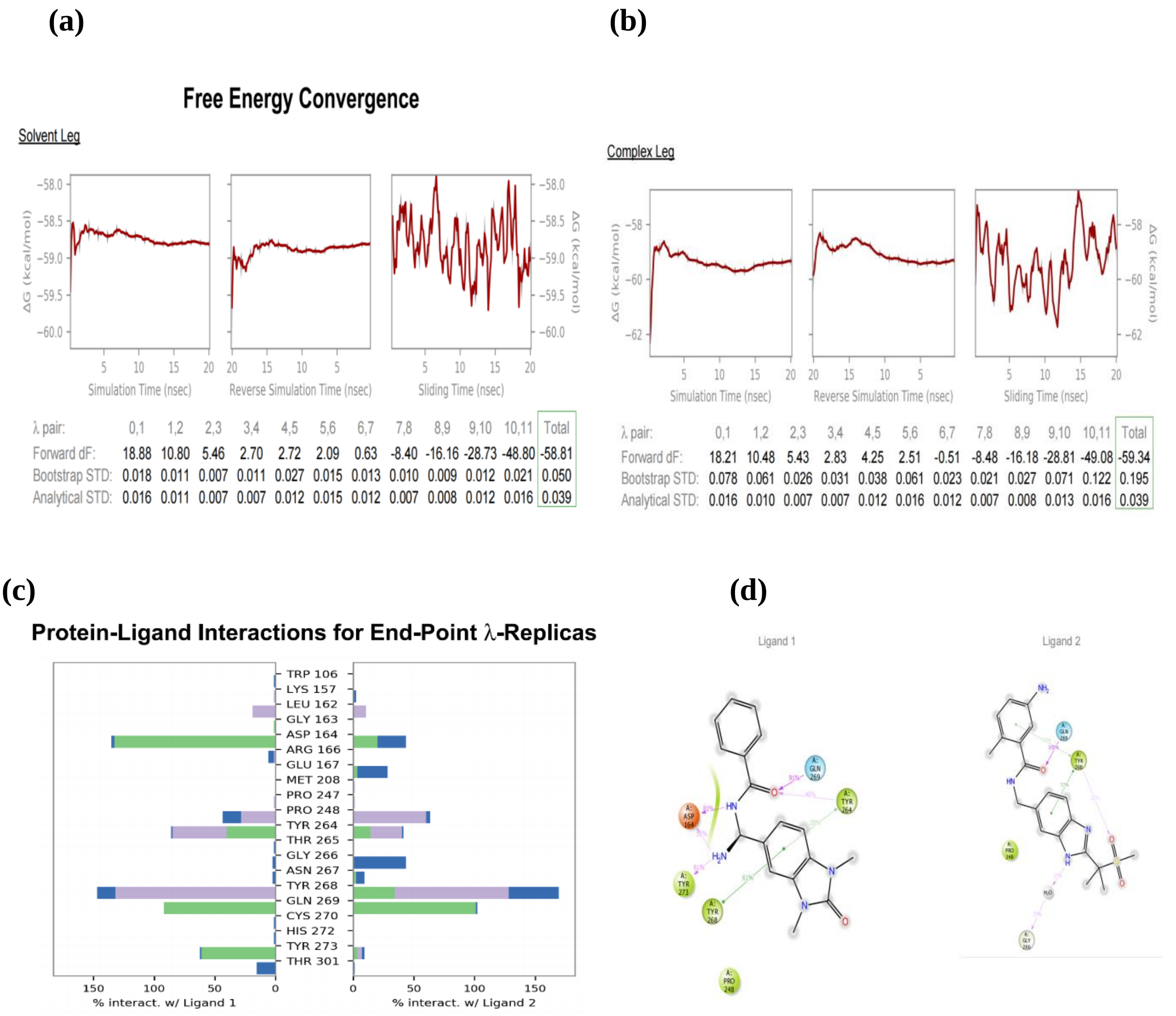

Solvent Leg:

λ pair:	0,1	1,2	2,3	3,4	4,5	5,6	6,7	7,8	8,9	9,10	10,11	Total
Forward dF:	18.88	10.80	5.46	2.70	2.72	2.09	0.63	-8.40	-16.16	-28.73	-48.80	-58.81
Bootstrap STD:	0.018	0.011	0.007	0.011	0.027	0.015	0.013	0.010	0.009	0.012	0.021	0.050
Analytical STD:	0.016	0.011	0.007	0.007	0.012	0.015	0.012	0.007	0.008	0.012	0.016	0.039

Complex Leg:

λ pair:	0,1	1,2	2,3	3,4	4,5	5,6	6,7	7,8	8,9	9,10	10,11	Total
Forward dF:	18.21	10.48	5.43	2.83	4.25	2.51	-0.51	-8.48	-16.18	-28.81	-49.08	-59.34
Bootstrap STD:	0.078	0.061	0.026	0.031	0.038	0.061	0.023	0.021	0.027	0.071	0.122	0.195
Analytical STD:	0.016	0.010	0.007	0.007	0.012	0.016	0.012	0.007	0.008	0.013	0.016	0.039

Figure S3. FEP+ simulation results for relative binding affinity prediction for the perturbation cycle of compound **5** (ligand 1) and compound **45** (ligand 2). **(a)** Free energy convergence of the perturbation cycle in solvent leg. **(b)** Free energy convergence of the perturbation cycle in complex leg. **(c)** Histogram depicting protein-ligand interactions for endpoint λ-replicas. **(d)** Ligand interaction diagram detailing the type of interactions observed between the two ligands and the receptor.

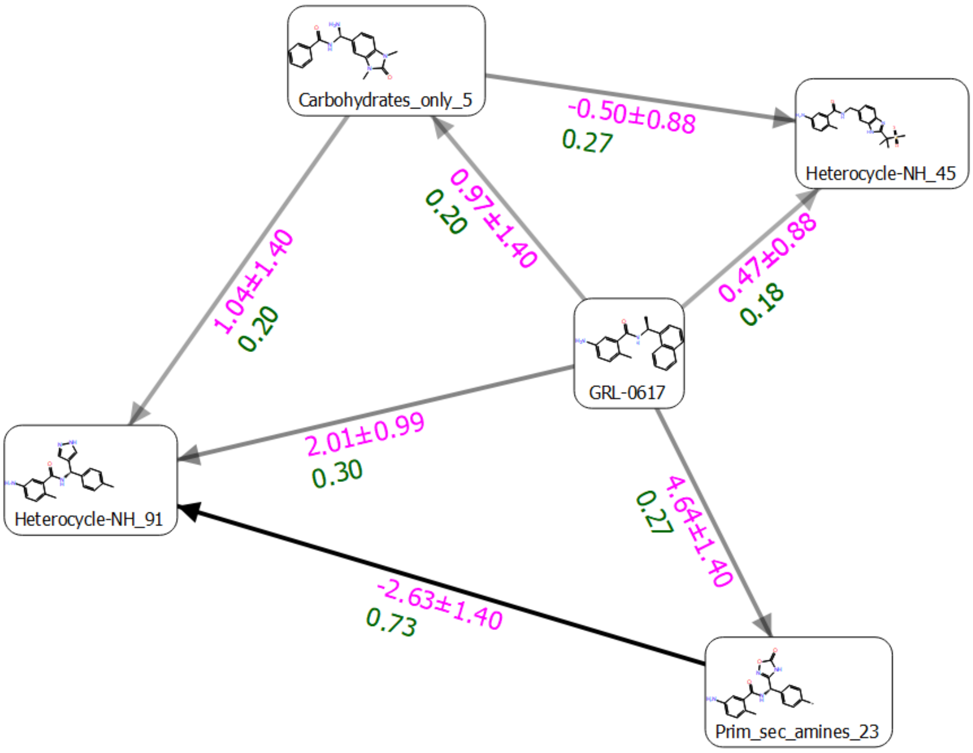

Figure S4. A circle closure perturbation map of design ideas and GRL-0617 detailing the difference in binding free energy, ΔΔG (pink) and its associated error estimate between two ligands in an edge/node, as well as their associated ligand similarities scores (green).

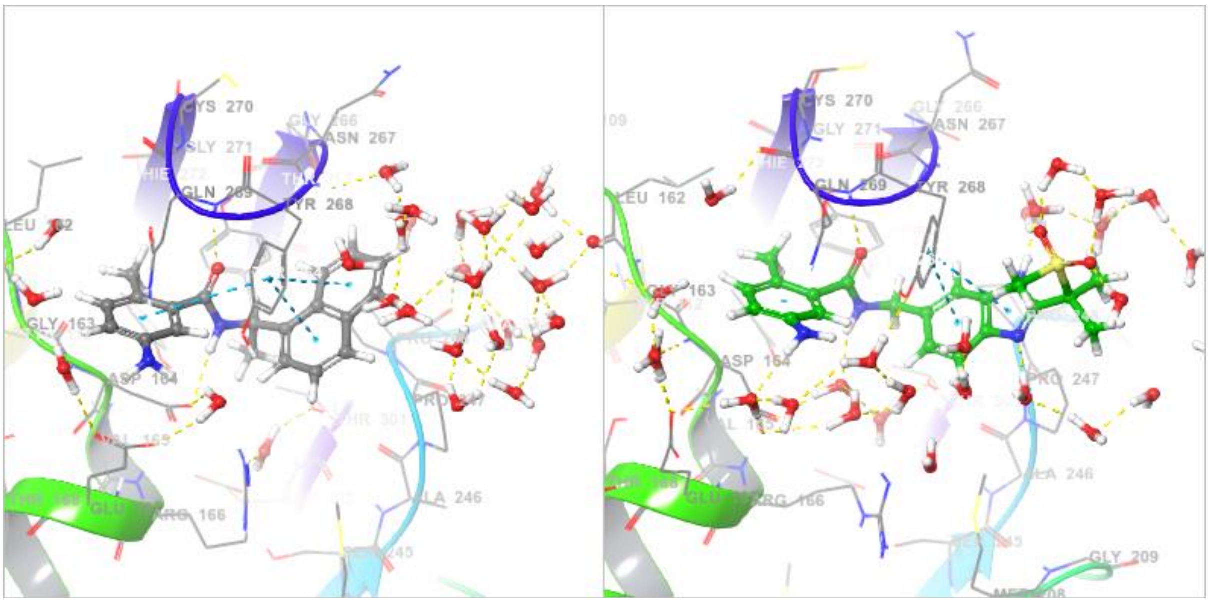

Figure S5. FEP+ node representation for GRL-0617 (grey carbons) on the left and compound **45** (green carbons) on the right frame in the active site cavity of PL^{pro} with favourable and unfavourable waters shown.

Table S1. Relative binding affinity prediction and its associated predicted error in kcal/mol of the design ideas and GRL-0617 as a reference compound against SARS-COV-2 PLpro using FEP+.

No.	Ligand	Estimated ΔG	Pred. ΔG kcal/mol	Pred. Error kcal/mol	Exp. ΔG kcal/mol	Exp. Error	Structure
1	Primary & secondary amines_23	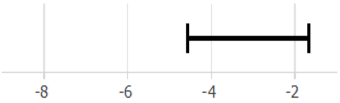	-3,11	1,45	N/A	N/A	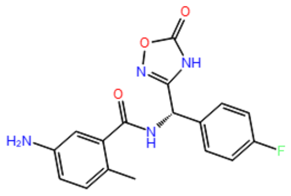
2	Carboxylates_only_5	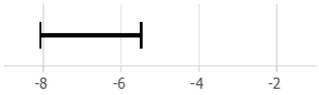	-6,78	1,30	N/A	N/A	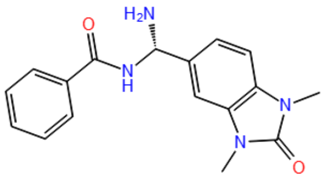
3	N-Heterocycles_91	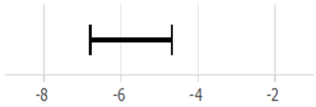	-5,74	1,06	N/A	N/A	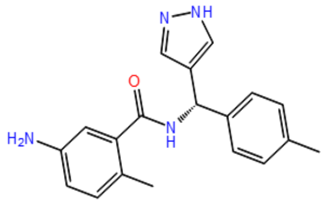

4	GRL-0617	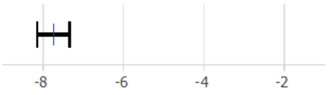	-7,75	0,40	-7,75	0	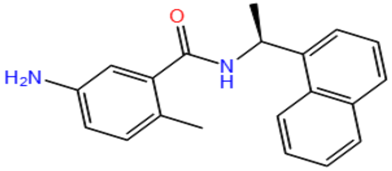
5	N-Heterocycles_45	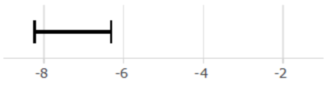	-7,28	0,96	N/A	N/A	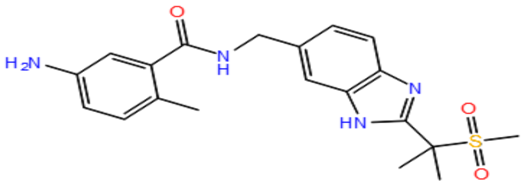
